# Class I Areas at Risk: Event-Based Nitrogen Deposition to a High-Elevation, Western Site

**DOI:** 10.1100/tsw.2001.271

**Published:** 2001-10-31

**Authors:** Mark W. Williams, Dave Manthorne

**Affiliations:** Institute of Arctic and Alpine Research and Department of Geography, University of Colorado, Boulder, USA

**Keywords:** nitrogen deposition, NADP, Class I areas, critical levels, power plants, mountains

## Abstract

Between June 1, 2000 and September 30, 2000, 32 precipitation events were sampled near Telluride, CO at an elevation of 3200 m. The wet deposition site was operated following protocols of the Atmospheric Integrated Research Monitoring Network (AIRMoN), a network of the National Atmospheric Deposition Network (NADP). Inorganic nitrogen deposition at the Telluride site of 1.41 kg ha during the study period was 25 to 50% higher than nearby NADP sites. In turn, nitrogen deposition at these NADP sites was similar to high-elevation sites in and near the Colorado Front Range that have been shown to be impacted by atmospheric deposition of inorganic nitrogen in wetfall. Power plant emissions are a likely source of a major portion of this elevated inorganic nitrogen in wetfall to the San Juan Mountains. Principal component analysis (PCA) shows that solutes formed by gases that are emitted from power plants were clustered tightly together, including nitrate, ammonium, sulfate, and chloride. Trajectory analysis, including both backward and forward trajectories, shows that the air masses that contributed to the precipitation events with high amounts of nitrogen deposition at the Telluride site passed directly over or near power plants. Our results suggest that Class I Wilderness Areas in and near the San Juan Mountains are at risk to ecosystem impairment at present rates of atmospheric deposition of inorganic nitrogen in wetfall. Deployment of proposed power plants to this area will likely increase the risk of degradation of resource values in nearby Class I areas. While these data were collected over a short time span, they indicate that establishment of an official AIRMoN site in the southwestern U.S. may be warranted.

## INTRODUCTION

Here we report on results from an Atmospheric Integrated Research Monitoring Network (AIRMoN)-type site operated in the Colorado Rocky Mountains. The research was prompted by results that show Class I areas of the Colorado Front Range are at risk to atmospheric deposition of nitrogen[1]. With funding from Region 8 of the EPA, we installed and operated an event-monitoring site during the summer of 2000, near Telluride, CO at an elevation of 3200 m. The NADP operates about 220 wet precipitation collectors throughout the continental U.S. Samples are collected and analyzed using the same protocols, so that precipitation chemistry may be compared among sites. Whereas the NADP was designed to characterize long-term trends in the chemical climate of the U.S., AIRMoN was designed to provide data with a greater temporal resolution. This short-term resolution is critical for (1) determining the effectiveness of emission controls mandated by the Clean Air Act, (2) evaluating the potential impacts of new sources of emissions on protected areas such as Class I areas, and (3) identifying source/receptor relationships in atmospheric models. Currently, there are nine wet AIRMoN sites, all located east of the Mississippi River and at elevations less than 1000 m. At present, there are no AIRMoN sites deployed to evaluate the potential impacts of new sources of emissions in Class I areas of the western U.S. Our site was not part of the official AIRMoN program, but was operated and maintained according to AIRMoN protocols except for sample analysis, as described below.

Emissions from upwind utilities fueled with local sub-bituminous coal are a potential source of elevated nitrogen deposition in wetfall to the San Juan Mountains of southern Colorado, and in particular to the headwaters of the San Miguel River. This research is particularly important because of the recent and ongoing discussions on adding new power plants in the western U.S. Here we report on our efforts to determine if the headwaters of the San Miguel River basin do receive elevated amounts of inorganic nitrogen in wetfall, and to evaluate the sources of precipitation events that contained higher amounts of inorganic nitrogen in wetfall. We installed and operated the event sampler site near Telluride in the headwaters of the San Miguel River during summer 2000 to:

1. Determine if the San Miguel drainage receives more atmospheric deposition of inorganic nitrogen in wetfall than nearby NADP sites at Molas Pass and Wolf Creek Pass;
2. Use the precipitation chemistry from the AIRMoN site to parameterize models such as HYSPLIT-4[2] to compute trajectories of air masses that may transport pollution;
3. Assess the possible consequences of additional water quality problems from (a) mercury deposition and (b) deployment of additional power plants; and
4. Evaluate the need for an official AIRMoN site in the Rocky Mountains.

## SITE DESCRIPTION

The event sampler was located at an elevation of 3200 m in Waterfall Canyon, about 15 km from the town of Telluride and its

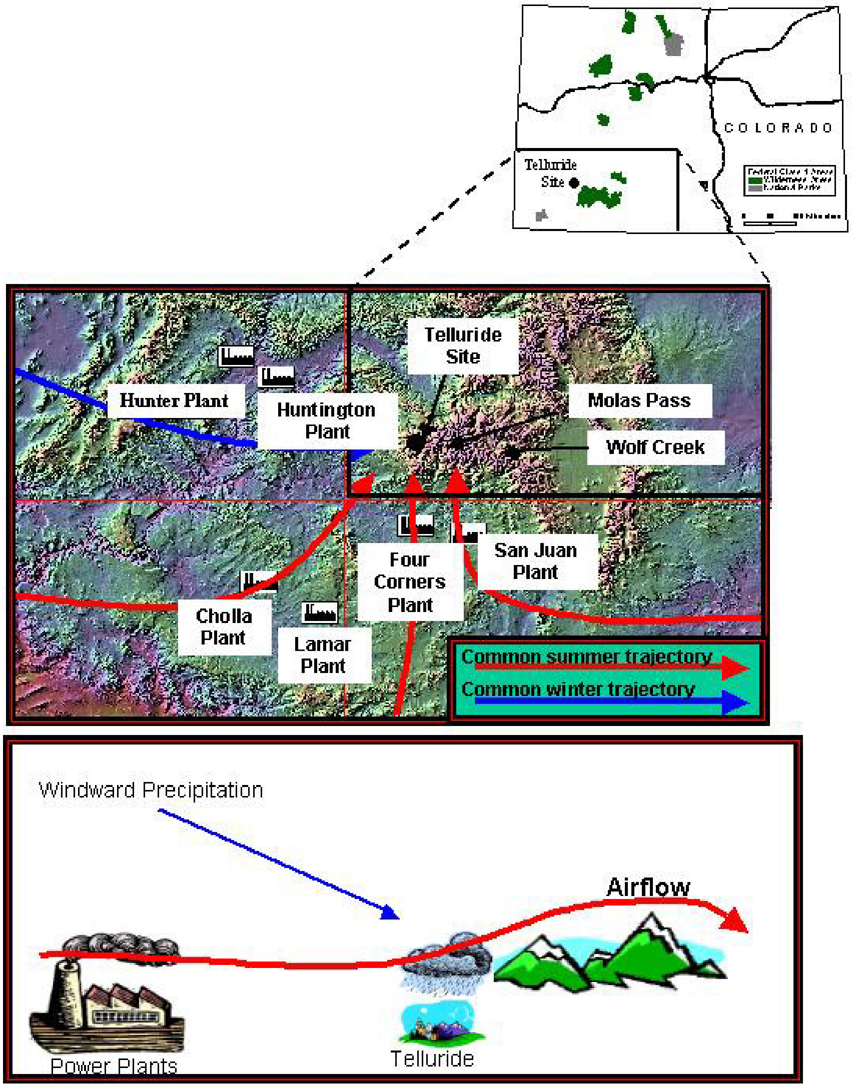

**FIGURE 1.** A digital elevation map showing the location of the event site near Telluride, nearby NADP sites at Molas Pass and Wolf Creek Pass, and major power plants in the region, along with Class I areas in the State of Colorado (NPS and USFS administrative units). The lower panel is a schematic of airflow patterns at the headwaters of the San Miguel River basin. As air masses cool adiabatically to lift over the first mountain barrier they encounter, precipitation is dropped on the windward side of the mountains. Since these air masses have passed over or near power plants, they are the first area where the pollution in air masses from power plants is scrubbed by precipitation.

associated skiing activities (Fig. 1). Waterfall Canyon was selected as the site for the study for the following reasons: (1) previous research has shown elevated amounts of nitrate in the streamwaters of Waterfall Canyon[3], (2) the location meets all NADP requirements for site selection, and (3) sample collection on an event basis was possible.

The location of the site in the headwaters of the San Miguel River basin is ideally suited to provide information on the potential impacts of increases in atmospheric deposition to Class I areas in Colorado if proposed power plants go online. Coal-fire power plants form an 180° arc from the northwest to southeast of the San Juan Mountains (Fig. 1). Class 1 areas are located throughout the region (Fig. 1), including the Weminuche Wilderness Area near the Telluride site. Additionally, there are several designated Wilderness Areas such as Lizard Head and Mt. Sneffels near the event collector that are not Class I areas yet contained pristine and important resource values. Our back- and forward-trajectory analyses will provide information on whether these power plants are potential sources of solutes in rainfall at the event site. Results from the event site are compared to results from NADP sites at Molas Pass (elevation 3249 m) and Wolf Creek Pass (3292 m) in the San Juan Mountains (Fig. 1).

## METHODS

### Installation and Operation

Samples were collected on an event basis during the summer of 2000. Measurement, data acquisition, and laboratory operations followed the procedures detailed in Miscellaneous Publication 188 by NADP, with the following exception: all samples were analyzed by the Institute of Arctic and Alpine Research (INSTAAR), University of Colorado. Samples were collected on an event basis. Cleaned buckets were changed if there was no precipitation event for 7 days. Collected samples were sent overnight express to the INSTAAR wet chemistry laboratory. At the laboratory, samples were analyzed within 48 h for major solutes, including pH, conductance, ANC, base cations, ammonium, anions, and Si.

INSTAAR participates in the NADP, operating two sites: CO02 (Niwot Saddle) and CO94 (Sugarloaf). We conducted quality analysis and quality assurance for ammonium and nitrate analysis at INSTAAR by analyzing splits of the NADP samples collected at sites CO02 and CO94. A paired-difference t-test between results from our laboratory and the NADP laboratory showed no significant difference for either ammonium or nitrate ($n = 67$; $p > 0.05$ for both N species)[4].

### Trajectory Analysis

We conducted backward and forward trajectories of deposition events with elevated amounts of inorganic nitrogen in wetfall to evaluate potential source areas. Trajectories were produced using National Oceanic and Atmospheric Administration (NOAA) HYbrid Single Particle Lagrangian Integrated Trajectories (HYSPLIT-4)[5]. HYSPLIT-4 has been used to investigate speciation and deposition of atmospheric mercury[6] and back trajectories of air masses[7], among many other uses. HYSPLIT-4 is a Lagrangian trajectory model that estimates particle movements in both forward and backward directions. Lagrangian methods are favored when point source emissions are restricting computations to a few grid points. Lagrangian models compute advection and dispersion components individually.

The meteorological data we used in the HYSPLIT-4 model is a 3-h archived data set called EDAS (Eta Data Assimilation System), covering the U.S.[8] The EDAS is an intermittent assimilation system consisting of successive 3-h forecasts on a 48-km grid. The 3-h analysis updates allow for the use of high frequency observations, such as wind profiler, NEXRAD, and aircraft data. NOAA's Air Resources Laboratory (ARL) saves the successive 3-h analysis twice a day to produce a continuous data archive. The 48-km data are interpolated to a 40-km Lambert Conformal Grid, which covers the continental U.S.

## RESULTS

### Precipitation Chemistry

Between June 1, 2000 and September 30, 2000, 32 precipitation events were sampled. The pH of precipitation ranged from 4.50 to 7.03, with a median value of 5.32. Ammonium concentrations ranged from 4.27 to 104.69 $\mu eql^{-1}$, with a median value of 22.4 $\mu eql^{-1}$. Nitrate concentrations ranged from 8.73 to 127.73 $\mu eql^{-1}$, with a median value of 32.8 $\mu eql^{-1}$. Sulfate concentrations varied from 10.33 to 79.62 $\mu eql^{-1}$ with a median value of 25 $\mu eql^{-1}$, a smaller range than for either $NH_4^+$ or $NO_3^-$ concentrations.

We used PCA to evaluate the relationship of solutes in precipitation and thus to better understand potential sources of the solutes. A correlation matrix was used so that each variable was given equal weight. In order to make the extracted components close to their orthogonal axes, Varimax rotation was applied[9]. The PCA chart is plotted using loadings (eigenvectors). The first two components have an eigenvalue greater than 1 and explain 81% of the total variance (Fig. 2). Sodium, chloride, sulfate, nitrate, ammonium, and potassium are clustered and characterize the first component. ANC, calcium, and silica are clustered and characterize the second component. The third cluster consists of hydrogen. Variables in the same cluster suggest a similar source (e.g., Baron[10]; Caine and Thurman[11]; Evans et al.[12]). Thus, sodium, chloride, sulfate, nitrate, ammonium, and potassium have a similar source that is different than the source of silica, ANC, calcium, and hydrogen. The strong mineral acids (nitrate, sulfate, and chloride) have a similar source, which may be power plant emissions of $SO_x$, $NO_x$, and related gasses. Ammonium is also produced as a byproduct of power plant emissions. However, local ranching and agricultural activities are an additional source of ammonium and may contribute to some unknown degree. The second cluster of ANC, calcium, and silica most likely represents local dust sources different than power plant emissions.

Inorganic nitrogen deposition at the event site was 1.41 kg $ha^{-1}$ during the study period (Fig. 3). To place these values in perspective, we compared inorganic nitrogen deposition at the event site to the nearby NADP mountain sites at Molas Pass and Wolf Creek Pass. Inorganic nitrogen deposition at Wolf Creek Pass of 0.79 kg $ha^{-1}$ was about half that of the event site. Deposition of inorganic nitrogen at Molas Pass of 1.06 kg $ha^{-1}$

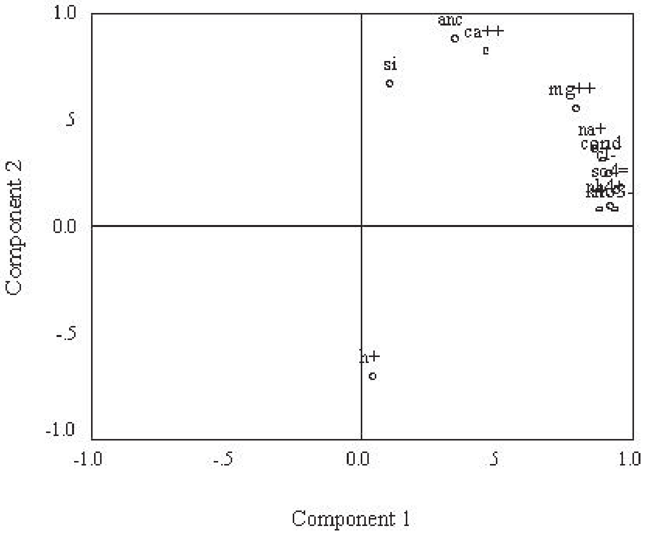

**FIGURE 2.** Principal component analysis of all solutes in precipitation at the event site. The strong acid anions and ammonium are clustered together, suggesting they are from the same source. Alkalinity and calcium show a different cluster, suggesting a different source area.

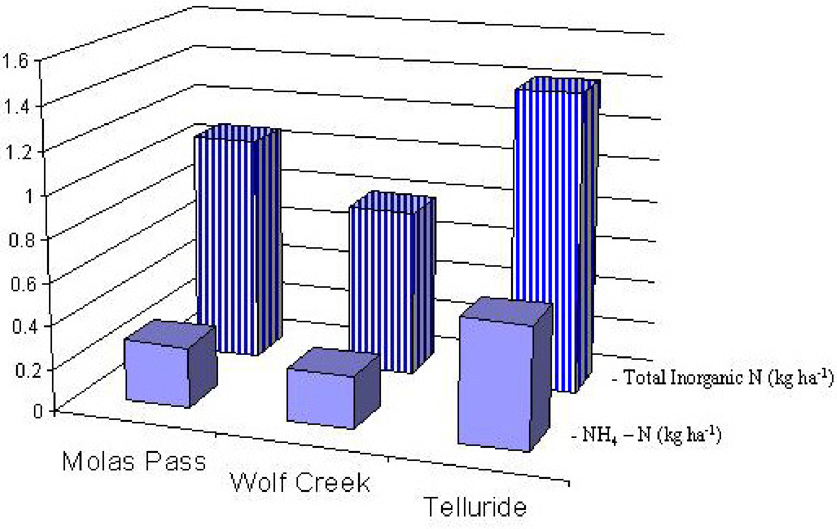

**FIGURE 3.** Ammonium-nitrogen and total inorganic nitrogen deposition in wetfall to the event site and comparison to nearby NADP sites at Wolf Creek Pass and Molas Pass for the period of study.

was 75% of the inorganic nitrogen in wetfall at the event site. Wetfall of inorganic nitrogen at the event site was 25 to 50% higher than nearby NADP sites.

Increased precipitation amount was not the cause of the increased loading of inorganic nitrogen in wetfall at the event site. Precipitation amounts at the three sites varied by less than 10%, ranging from 19.3 mm at Wolf Creek Pass to 21.8 mm at the event site. Deposition of nitrate-nitrogen in wetfall at the event site of 0.84 kg $ha^{-1}$ was somewhat greater than the 0.78 kg $ha^{-1}$ at Molas Pass and 0.54 kg $ha^{-1}$ at Wolf Creek Pass. Ammonium-nitrogen deposition in wetfall at the event site of 0.57 kg $ha^{-1}$ was about twice the ammonium deposition at either Molas Pass (0.29 kg $ha^{-1}$) or Wolf Creek Pass (0. 25) (Fig. 3).

Higher ammonium concentrations appear to be the primary factor in the larger amounts of inorganic nitrogen deposition in wetfall at the event site relative to Molas Pass and Wolf Creek Pass. The VWM concentrations of nitrate were within about 20% at each site, ranging from 23 µeq$l^{-1}$ at Wolf Creek Pass to 31 µeq$l^{-1}$ at Molas Pass. However, VWM concentrations of ammonium of 20 µeq$l^{-1}$ at the event site were about twice the concentrations of 11 µeq$l^{-1}$ at Molas Pass and Wolf Creek Pass.

There is one caveat in this analysis that inorganic nitrogen deposition is greater at the event site than Wolf Creek Pass and Molas Pass. The increase in loading of inorganic nitrogen at the event site was primarily driven by more ammonium at that site. The AIRMoN protocol utilized at the event site may result in higher amounts of ammonium recovery at the event site compared to regular NADP sites such as Molas Pass and Wolf Creek Pass. The reason is because ammonium is a volatile compound and some ammonium may be lost from the collector bucket at NADP sites because they are sampled on a weekly basis[13].

## Trajectory Analysis

We conducted five back-trajectory analyses using HYSPLIT-4 on precipitation events with the five highest amounts of inorganic nitrogen deposition (Table 1). The amount of water in the five events ranged from 14 to 26 mm. Inorganic nitrogen deposition ranged from 0.11 to 0.16 kg ha$^{-1}$. Source direction of air masses ranged over approximately 180°, from about 160° on September 6th to 340° on September 24th. All air masses passed either directly over or very near power plants (Table 1).

**TABLE 1**
**Back-Trajectory Analyses using HYSPLIT-4**

| Date | Precip Amount (mm) | N Deposition (kg ha$^{-1}$) | Wind Direction | Power Plants |
|---|---|---|---|---|
| 19-Aug | 23 | 0.11 | Westerly | Mohave, Blundell |
| 25-Aug | 26 | 0.16 | Southerly | San Juan, Four Corners |
| 6-Sep | 19 | 0.13 | South-westerly | Cholla, Four Corners |
| 22-Sep | 18 | 0.14 | Westerly | Mohave, Blundell |
| 24-Sep | 14 | 0.12 | North-westerly | Huntington, Hunter |

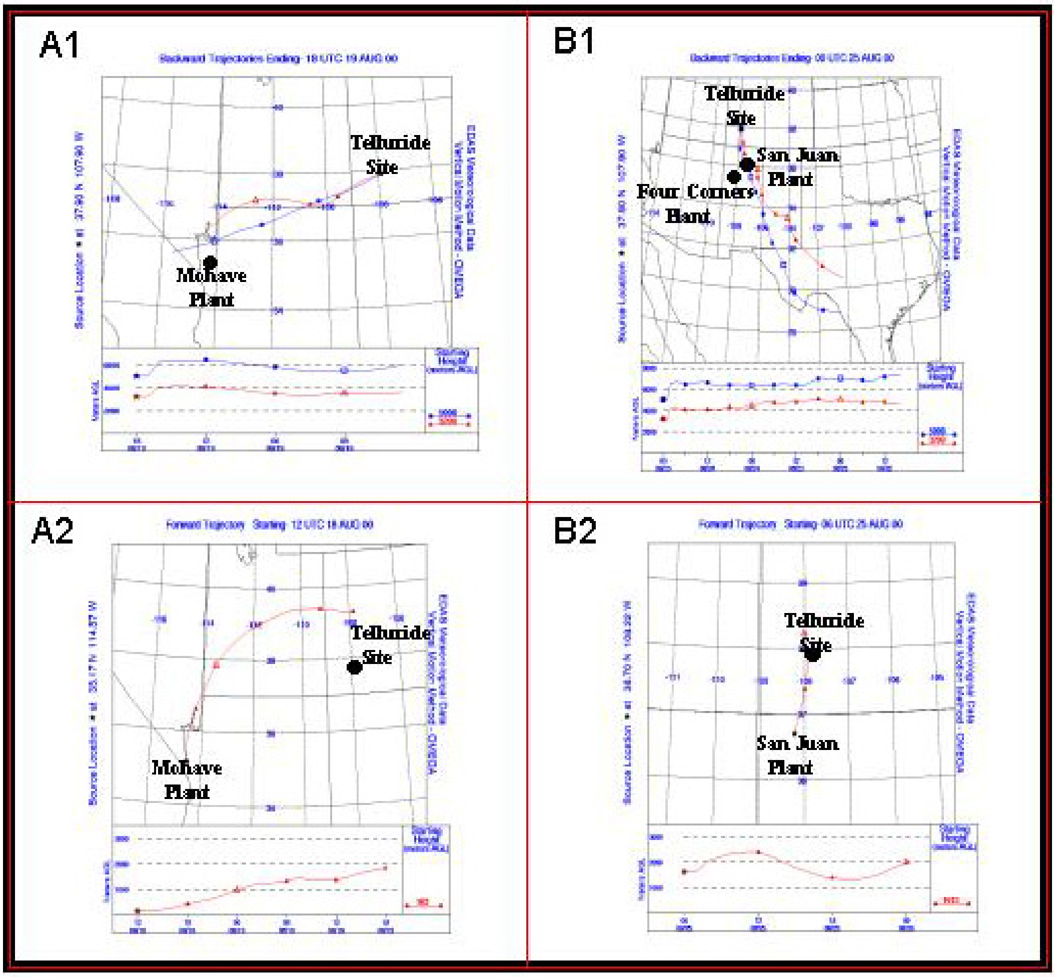

**FIGURE 4.** (A) Backward- and forward-trajectory analyses using HYSPLIT-4 for August 18, 2000. Both trajectories shows that air masses passed over the Mojave Power Plant before reaching the event site. (B) Backward- and forward-trajectory analyses using HYSPLIT-4 for August 25, 2000. Air masses over the San Juan power plant reached Telluride and resulted in large amounts of inorganic nitrogen in wetfall at the event site.

Individual trajectories are illustrated in Fig. 4A1 and 4B1. We ran the back trajectories at two heights, 5000 and 3200 m. In general, there was little difference in back trajectories at the two heights for the first 24 h, except for the event on September 24th. At periods longer than 24 h, the back trajectories at the two heights showed increasing divergence.

We also ran forward trajectories from power plants for the same precipitation events. A measure of the integration error may be obtained by computing a backward trajectory from the end-point position of its forward counterpart. The forward trajectory for August 18th shows emissions from the Mohave power plant going within 90 km of the event site on August 19th, which the back trajectory analysis showed going over the event site (Fig. 4A2). Similarly, a forward trajectory on August 25, 2000 from the San Juan power plant shows the airmass directly over the event site, similar to the back-trajectory analysis (Fig. 4B2). For all five cases, forward trajectories were within 90 mi of the backward trajectories.

## DISCUSSION

### Class I Areas

Our results suggest that Class I areas in and near the San Juan Mountains are at risk to ecosystem impairment at present rates of atmospheric deposition of inorganic nitrogen in wetfall. Annual deposition of inorganic nitrogen to Wolf Creek Pass of about 3 kg $ha^{-1}$ $year^{-1}$ since 1993 is similar to high-elevation sites in and near the Colorado Front Range that have been shown to be impacted by atmospheric deposition of inorganic nitrogen in wetfall[1,14,15]. Atmospheric deposition of inorganic nitrogen to the San Juan Mountains is high relative to other subalpine/ alpine sites such as the Sierra Nevada[16,17].

Deployment of new power plants in the future will likely increase the risk of degradation of Class I resources in and near the San Juan Mountains. According to the recent biennial report to Congress by the National Acid Precipitation Assessment Program[18], the Rocky Mountain region is particularly susceptible to impairment of ecosystem function from increasing levels of atmospheric deposition of nitrogen because of extensive areas of exposed and unreactive bedrock, rapid hydrologic flushing rates during snowmelt, limited extent of vegetation and soils, and short growing seasons. The recent measurement in the Colorado Rockies of the most acidic snow found west of the 100th meridian[19], reports of nitrogen saturation in the Colorado Front Range[14,20], and episodic acidification of headwater catchments[21,22] add urgency to understanding how increases in nitrogen deposition may be changing water quality in Class I areas in and near the San Juan Mountains.

### Windward Location and Power Plant Emissions

Power plant emissions are the likely source of at least some of this elevated inorganic nitrogen in wetfall to the San Juan Mountains. PCA analysis shows that solutes produced from gases that are emitted from power plants were clustered tightly together, including nitrate, ammonium, sulfate, and chloride. Trajectory analysis, including both backward and forward trajectories, shows that the air masses that contributed to the precipitation events with high amounts of nitrogen deposition at the event site passed directly over or near power plants.

The relatively high rates of inorganic nitrogen loading in wetfall to both the NADP sites at Wolf Creek Pass and Molas Pass as well as our event site may be due to a combination of orographic precipitation and windward location with respect to pollution sources. Increased precipitation with increasing elevation leading to increased deposition of pollutants in wetfall has been reported in the northeast U.S.[23], Great Smoky Mountains[24], and the Colorado Front Range[1]. In combination with orographic effects, the Telluride sampling site and the NADP site at Wolf Creek Pass are on or near the windward side of mountains that receive airflow from power plants during the summer (Fig. 1). As air masses cool adiabatically to lift over the first mountain barrier they encounter, precipitation is dropped on the windward side of the mountains. Since these air masses have passed over or near power plants, they are the first area where the pollution in air masses from power plants is scrubbed by precipitation. Moreover, these precipitation events have larger amounts of water because they are occurring on the windward side of mountains. Thus loading of inorganic nitrogen in wetfall is particularly high on the windward side of these mountain areas that are downwind from power plants.

These results suggest that there is reason to be concerned about resource damage from atmospheric deposition of inorganic nitrogen in wetfall in and near the San Juan Mountains. While we evaluated only inorganic nitrogen for this study, there is the possibility that other pollutants may be causing resource damage. For example, five reservoirs in Colorado appeared on the EPA section 303(d) list of impaired water bodies because of elevated mercury content in fish. Four of the five reservoirs are in southwestern Colorado downwind of coal-fired power plants. Establishment of an official AIRMoN site in the western U.S. may be warranted.

## ACKNOWLEDGMENTS

Thanks to Leigh Sullivan and the citizens of Ophir for collecting the samples. Thanks to Chris Seibold for conducting the chemical analyses at INSTAAR and Fengjing Liu for the PCA analysis. Funding assistance was provided by San Miguel County, EPA Region 8, and National Science Foundation grant DEB 9810218 to the NWT LTER site. The manuscript was greatly improved by the comments from T. Meixner, M. Losleben, and an anonymous reviewer.

| | | |
|---|---|---|
| Received: | July | 10, 2001 |
| Revised: | October | 1, 2001 |
| Accepted: | October | 5, 2001 |
| Published: | October | 31, 2001 |
